# Impact of Surgical Approach on Surgical Resection Quality in Mid- and Low Rectal Cancer, A Bayesian Network Meta-Analysis

**DOI:** 10.3389/fonc.2021.699200

**Published:** 2021-08-11

**Authors:** Xiaojie Wang, Zhifang Zheng, Qian Yu, Waleed M. Ghareeb, Xingrong Lu, Ying Huang, Shenghui Huang, Shuangming Lin, Pan Chi

**Affiliations:** ^1^Department of Colorectal Surgery, Union Hospital, Fujian Medical University, Fuzhou, China; ^2^Department of Pathology, Union Hospital, Fujian Medical University, Fuzhou, China; ^3^Department of Gastrointestinal and Anal Surgery, Longyan First Hospital, Affiliated to Fujian Medical University, Longyan, China

**Keywords:** robotic, transanal, laparoscopic, rectal cancer, quality of surgical resection

## Abstract

**Aim:**

To evaluate the evidence concerning the quality of surgical resection in laparoscopic (LapTME), robotic (RobTME) and transanal (TaTME) total mesorectal excision for mid-/low rectal cancer.

**Methods:**

A systematic literature search of the PubMed, EMBASE and Cochrane Central Register of Controlled Trials databases was performed. A Bayesian network meta-analysis was utilized to compare surgical resection involved in these 3 surgical techniques by using ADDIS software. Rates of positive circumferential resection margins (CRMs) were the primary endpoint.

**Results:**

A total of 34 articles, 2 randomized clinical trials (RCTs) and 32 non-RCTs, were included in this meta-analysis. Pooled data showed CRM positivity in 114 of 1763 LapTME procedures (6.5%), 54 of 1051 RobTME procedures (5.1%) and 60 of 1276 TaTME procedures (4.7%). There was no statistically significant difference among these 3 surgical approaches in terms of CRM involvement rates and all other surgical resection quality outcomes. The incomplete mesorectal excision rates were 9.6% (69/720) in the LapTME group, 1.9% (11/584) in the RobTME group and 5.6% (45/797) in the TaTME group. Pooled network analysis observed a higher but not statistically significant risk of incomplete mesorectum when comparing both LapTME with RobTME (OR = 1.99; 95% CI = 0.48-11.17) and LapTME with TaTME (OR = 1.90; 95% CI = 0.99-5.25). By comparison, RobTME was most likely to be ranked the best or second best in terms of CRM involvement, complete mesorectal excision, rate of distal resection margin (DRM) involvement and length of DRMs. In addition, RobTME achieved a greater mean tumor distance to the CRM than TaTME. It is worth noting that TaTME was most likely to be ranked the worst in terms of CRM involvement for intersphincteric resection of low rectal cancer.

**Conclusion:**

Overall, RobTME was most likely to be ranked the best in terms of the quality of surgical resection for the treatment of mid-/low rectal cancer. TaTME should be performed with caution in the treatment of low rectal cancer.

## Introduction

Total mesorectal excision (TME) remains the leading surgical approach in the treatment of patients with mid- and low rectal cancer (1). The feasibility of laparoscopic TME (LapTME) has been assessed in several studies and has been widely practiced as an alternative to open surgery in the treatment of mid-/low rectal cancer. This procedure has been found to be oncologically safe and associated with minimally invasive advantages, such as less pain, a shorter hospitalization time, and faster bowel function return (2). However, achieving high-quality TME dissection still might be technically demanding even by experts, especially for tumors in the lower two-thirds of the rectum or for bulky tumors in a narrow, irradiated deep pelvis during laparoscopic operations. The innate limitations associated with laparoscopic TME include the use of rigid instruments, the limited range of motion, the loss of dexterity, fixed trocar positions and the limited view in the narrow, deep pelvic cavity. Two randomized studies [ALaCaRT trial (3) and ACOSOG Z6051 trial (4)] on laparoscopic and open surgeries for the treatment of rectal cancer raised concerns regarding the quality of oncological resection, highlighting the risk of positive circumferential resection margins (CRMs) and incomplete mesorectal excision.

The introduction of two other minimally invasive surgical approaches, robotic (RobTME) and transanal total mesorectal excision (TaTME), for mid-/low rectal cancer surgical treatment has appeared to overcome some of the technical difficulties of laparoscopy (5, 6). The robotic system provides greater maneuverability by enabling surgeons to control wrist motion during the use of endoscopic instruments with high-definition three-dimensional steady vision. The transanal approach to TME was also developed with the aim of improving distal mesorectal dissection, which is the most technically challenging part of transabdominal LapTME, by improving visibility and access to the dissection planes deep in the lower pelvic cavity.

To date, two network meta-analyses comparing these 3 surgical techniques in rectal cancer have been published (7, 8). However, the results were conflicting in terms of the quality of surgical resection, which was measured using CRMs, mesorectal quality, and distal resection margins (DRMs) (9, 10). The first network meta-analysis performed by Simillis et al. (7) demonstrated a decreased rate of positive CRMs in TaTME compared to LapTME, which was in contrast with the results of the second network meta-analysis by Rausa et al. (8) To our knowledge, no prior studies have compared the quality of surgical resection of these three surgical approaches for mid-/low rectal cancer treatment. Therefore, we performed an updated network meta-analysis of the latest and most convincing evidence to evaluate the quality of surgical resection of these 3 minimally invasive surgical techniques for mid-/low rectal cancer.

## Materials and Methods

### Data Sources and Searches

The present study was designed according to the Preferred Reporting Items for Systematic Reviews and Meta-analysis (PRISMA) guidelines (11). A systematic literature search of the PubMed, EMBASE and Cochrane Central Register of Controlled Trials databases was performed up to June 2019. The ClinicalTrials.gov registry (https://clinicaltrials.gov/) was also considered. Specific research equations were formulated for each database using the following search terms: rectal cancer, rectal carcinoma, surgery, total mesorectal excision, laparoscopy, laparoscopic surgery, transanal total mesorectal excision, TaTME, and robotic surgery. Moreover, the references cited in relevant review articles were cross-checked to identify additional studies.

### Inclusion and Exclusion Criteria

We evaluated the checked studies against the following criteria:

1. Population: patients with mid-/low rectal cancer.
2. Intervention: TME.
3. Comparator: at least two of the methods for the treatment of mid-/low rectal cancer (LapTME, RobTME, and TaTME) were compared.
4. Outcome measure: pathological outcomes.
5. Study design: randomized clinical trials (RCTs) or nonrandomized comparative studies (non-RCTs).

All reviews, comments, case reports, and expert opinions were excluded. Duplicates were excluded.

### Data Extraction and Quality Assessment

The details of the included studies were extracted from the electronic databases independently by two investigators. Disagreements were resolved by joint review of the studies to reach consensus. The following data were obtained: characteristics of the studies, such as first author name, publication year, study time, surgical treatments, and number of each intervention; demographic characteristics of the participants; and details of the pathological outcomes, including CRM involvement, tumor distance to the CRM, length of DRMs, positive DRMs, mesorectal quality (complete, near complete and incomplete mesorectum), and harvested lymph nodes. The quality of the studies included in this systematic review was assessed independently by the same reviewers with Jadad scores (12) for RCTs and the Newcastle-Ottawa Scale (NOS) (13) for nonrandomized comparative studies.

### Outcomes

The rate of positive CRMs was the primary endpoint, and tumor distance to the CRM, length of DRMs, positive DRM rate, mesorectal quality (complete, near complete and incomplete mesorectum) and harvested lymph nodes were the secondary endpoints. A positive CRM was defined when the tumor was located 1 mm or less from the CRM (14). The quality of mesorectal excision was evaluated using the Quirke classification (9). Specifically, a complete mesorectum was defined as an intact mesorectum with only minor irregularities of a smooth mesorectal surface. No defect is deeper than 5 mm. Nearly complete mesorectum was defined as moderate bulk to the mesorectum, but at no site is the muscularis propria visible. Incomplete mesorectum was defined as little bulk to mesorectum with defects down onto muscularis propria (9).

### Statistical Analysis

We calculated the odds ratio (OR) and weighted mean difference (WMD) with a 95% confidence interval (CI) for dichotomous and continuous variables. If studies only reported median values or range values, the original data were transformed into forms suitable for meta-analysis using the algorithms proposed by Hozo et al. (15) We performed the multi-treatment network meta-analysis within a Bayesian framework with the Markov Chain Monte Carlo simulation. All data were calculated by using the Aggregate Data Drug Information System (ADDIS) v1.0 and STATA (version 15.0; StataCorp, College Station, TX). The parameters for the network meta-analysis in the ADDIS were as follows: the number of chains, 4; tuning iterations, 20,000; simulation iterations, 50,000; thinning interval, 10; inference samples, 10,000; and variance scaling factor, 2.5. The convergence of the model was judged by the potential scale reduction factor (PSRF) (16); a PSRF closer to 1 indicated better convergence.

Traditional pairwise meta-analysis of direct comparisons was performed using STATA. Statistical heterogeneity between studies was assessed with I^2^ statistics. Values of I^2^ above 25% and lower than 25% were regarded as heterogeneity and no heterogeneity, respectively (17). A random-effects model was used to incorporate direct data into a single comparison if heterogeneity existed (I^2^ > 25%). A fixed-effects model was used for variables with I^2^ values lower than 25%.

For the closed-loop comparisons, the consistency test between direct comparisons and indirect estimated comparisons was judged using node-splitting analysis. A consistency model was used when the P value >0.05 in the node-splitting analysis; otherwise, the inconsistency model was used (18). Finally, ranking probabilities were calculated for the results of each treatment under different endpoints to provide the basis for alternative selection.

## Results

### Identification of Studies

**Figure 1:**
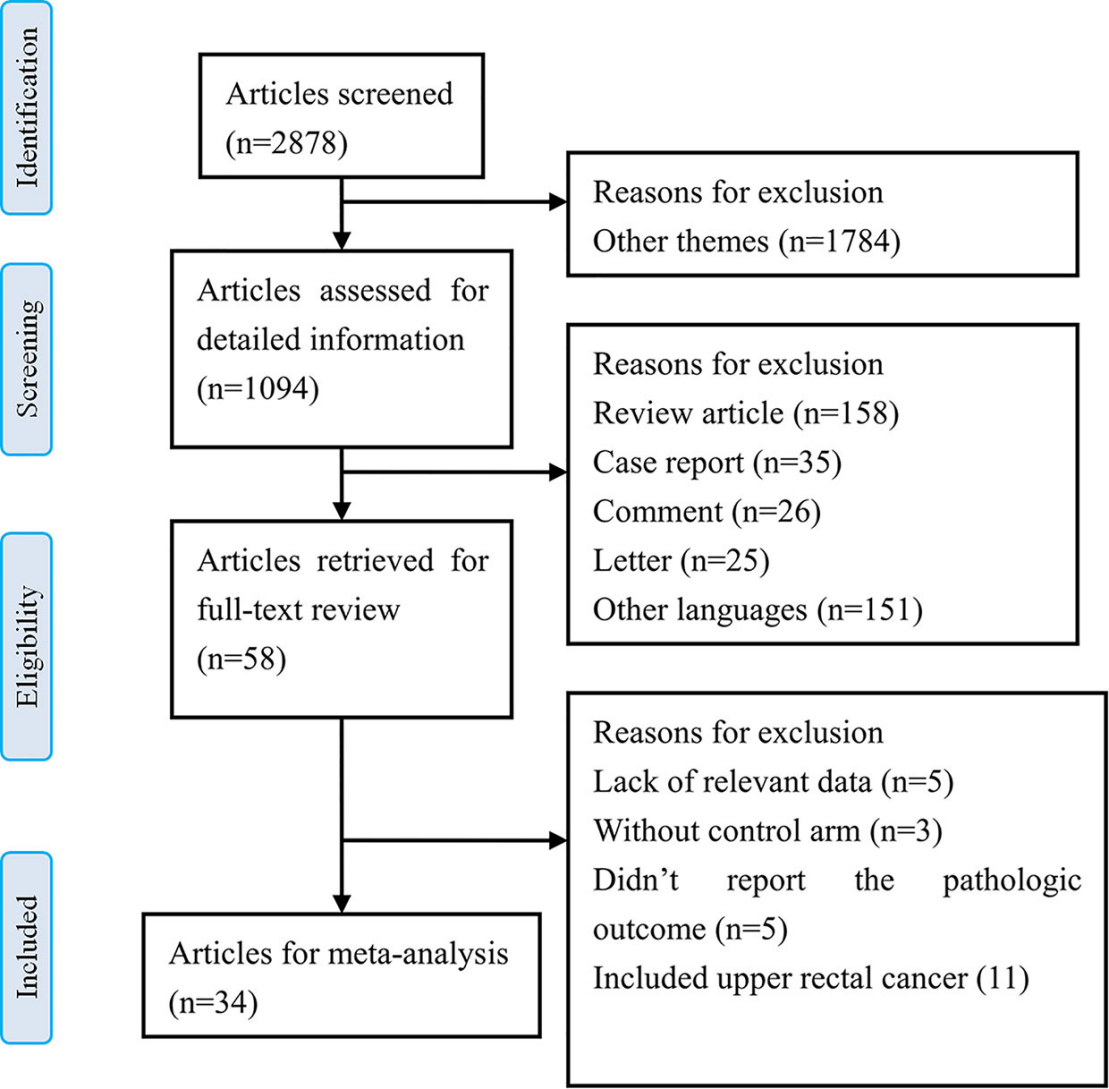
Flow chart for the literature search and study selection.

The results of the literature search identified 2878 articles for initial screening based on the titles. Among them, 1094 articles were imported for detailed information based on the abstracts. Of these, 58 articles were retrieved for full-text review, and among them, 24 studies were excluded based on the selection criteria. Finally, we included 34 relevant articles that were reviewed for meta-analysis (5, 6, 19–50). There were 2 RCTs and 32 nRCTs. A flow chart for the literature search and study selection is shown in **Figure 1**.

### Characteristics of the Included Studies

**Figure 2:**
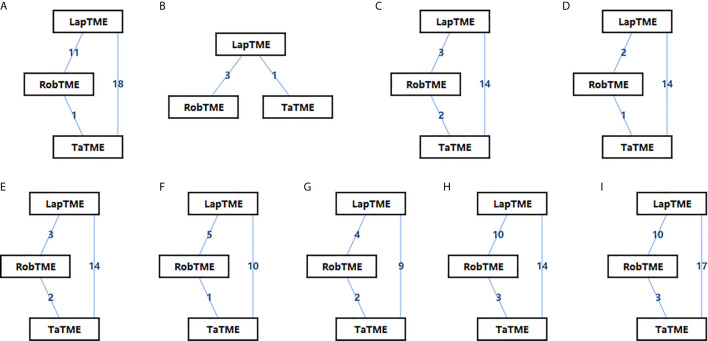
Network diagrams of the eligible studies. **(A)** CRM involvement; **(B)** CRM involvement for ISR; **(C)** Complete mesorectum; **(D)** Near complete mesorectum; **(E)** Incomplete mesorectum; **(F)** DRM involvement; **(G)** Tumor distance to the CRM; **(H)** DRM distance; **(I)** Harvested lymph nodes.

The characteristics of the included studies are summarized in **Table 1**. Of the included studies, 26 of 32 (80%) non-RCTs were of high quality (NOS score ≥ 7), and the other 2 RCTs were of medium quality (Jadad score = 3). In total, 4429 patients with mid-/low rectal cancer were included in the study: 1856 patients in the LapTME group, 1211 patients in the RobTME group, and 1362 patients in the TaTME group. Among them, there were more male (68.1%) than female patients (31.9%). The mean age varied from 54 to 70 years, and the mean body mass index (BMI) ranged from 21.4 kg/m^2^ to 29.0 kg/m^2^. The mean tumor distance from anal verge varied from 1.5 to 8.0 cm. Studies by JS Park et al. (24), Kuo et al. (21), SY Park et al. (20) and Kanso et al. (30) included only low rectal cancer patients who underwent intersphincteric resection (ISR). The connection between each surgical approach was analyzed, and each square reflecting the surgical approach and two squares linked together by an edge showed the number of studies comparing the two corresponding surgical techniques directly (**Figure 2**).

**Table 1:** Characteristics of included studies.

Study	Year	Study year	Study design	Group			Gender (male, %)						Mean age			Mean BMI			Tumor distance from anal verge (cm)			CRT (n, %)						Quality score&
				Lap	Rob	Ta	Lap		Rob		Ta		Lap	Rob	Ta	Lap	Rob	Ta	Lap	Rob	Ta	Lap		Rob		Ta		
Bianchi (5)	2010	2008-2009	Non-RCT	25	25		17	(68%)	18	(72%)			62.0	69.0		26.5	24.6		NA	NA		10	(40%)	13	(52%)			7
Baek (19)	2013	2007-2010	Non-RCT	37	47		28	(76%)	31	(66%)			61.8	58.0		23.4	23.4		5.5	4.4		12	(32%)	20	(43%)			9
SY Park (20)	2013	2008-2011	Non-RCT	40	40		25	(63%)	28	(70%)			63.6	57.3		24.3	23.9		3.6	3.4		20	(50%)	32	(80%)			6
Kuo (21)	2014	2009-2013	Non-RCT	28	36		17	(61%)	21	(58%)			54.9	55.9		NA	NA		3.7	3.8		28	(100%)	28	(78%)			6
Denost (22)	2014	2008-2012	RCT	50		50	32	(64%)			37	(74%)	63.0		64.0	25.6		25.1	4.0		4.0	44	(88%)			40	(80%)	3#
Velthuis (23)	2014	2012-2013	Non-RCT	25		25	18	(72%)			18	(72%)	65.0		64.0	25.0		27.0	6.0		8.0	25	(100%)			25	(100%)	9
JS Park (24)	2015	2008-2011	Non-RCT	106	106		71	(67%)	75	(71%)			61.7	59.6		23.8	24.3		3.3	3.2		60	(57%)	68	(64%)			9
Serin (25)	2015	2005-2013	Non-RCT	65	14		65	(100%)	14	(100%)			57.0	54.0		26.0	24.7					65	(100%)	14	(100%)			8
Yoo (26)	2015	2006-2011	Non-RCT	26	44		19	(73%)	35	(80%)			60.5	59.8		21.4	24.1		3.7	3.2		7	(27%)	24	(55%)			6
Chen (27)	2015	2013-2015	Non-RCT	100		50	76	(76%)			38	(76%)	58.3		57.3	24.6		24.2	6.7		5.8	100	(100%)			50	(100%)	7
De’Angelis (28)	2015	2008-2014	Non-RCT	32		32	21	(66%)			21	(66%)	67.1		64.9	24.5		25.1	3.7		4.0	23	(72%)			27	(84%)	9
Fernandez-Hevia (29)	2015	2011-2013	Non-RCT	37		37	22	(59%)			24	(65%)	69.5		64.5	25.1		23.7	NA		NA	21	(57%)			27	(73%)	8
Kanso (30)	2015	2005-2013	Non-RCT	34		51	26	(76%)			36	(71%)	59.0		59.0	24.0		24.0	1.8		1.6	28	(82%)			43	(84%)	9
Perdawood (31)	2015	2013-2015	Non-RCT	25		25	19	(76%)			19	(76%)	70.0		70.0	26.0		28.0	8.0		8.0	4	(16%)			7	(28%)	8
Bedirli (32)	2016	2013-2015	Non-RCT	28	35		19	(68%)	24	(69%)			60.4	64.7		23.2	24.7		NA	NA		28	(100%)	35	(100%)			8
Feroci (33)	2016	2004-2014	Non-RCT	58	53		42	(72%)	27	(51%)			66.0	66.0		24.6	24.6		8.0	8.0		25	(43%)	26	(49%)			6
Law (34)	2016	2008-2015	Non-RCT	171	220		97	(57%)	148	(67%)			67.0	65.0		24.6	24.9		8.0	7.0		50	(29%)	91	(41%)			6
Lim (35)	2016	2006-2010	Non-RCT	64	74		46	(72%)	50	(68%)			65.8	65.1		22.7	23.4		6.7	5.3		64	(100%)	74	(100%)			9
Chouillard (36)	2016	2011-2014	Non-RCT	15		18	7	(47%)			6	(33%)	57.8		55.4	29.0		27.1	NA		NA	12	(80%)			14	(78%)	8
Lelong (37)	2016	2008-2013	Non-RCT	38		34	22	(58%)			23	(68%)				24.2		24.0				35	(92%)			30	(88%)	9
Marks (38)	2016	2012-2014	Non-RCT	17		17	NA				NA		60.0		59.0	25.9		26.4	NA		NA	NA				NA		8
Rasulov (39)	2016	2013-2016	Non-RCT	23		22	14	(61%)			11	(50%)	60.0		56.0	26.0		26.0	7.0		6.5	19	(83%)			19	(86%)	7
Kim (40)	2017	2012-2015	RCT	73	66		52	(63%)	51	(61%)			59.7	60.4		23.6	24.1		NA	NA		58	(79%)	51	(77%)			3#
Perez (41)	2017	2013-2016	Non-RCT		60	55			44	(73%)	40	(73%)		NA	NA		25.8	24.9		NA	NA			42	(70%)	35	(64%)	8
Chang (6)	2017	2014-2017	Non-RCT	23		23	13	(57%)			13	(57%)	62.9		62.4	25.0		25.8	5.9		4.3	14	(61%)			8	(35%)	9
Perdawood2 (42)	2017	2015-2017	Non-RCT	100		100	69	(69%)			72	(72%)	66.9		67.3	25.4		25.7	7.8		7.5	27	(27%)			18	(18%)	8
Lee (43)	2018	2011-2017	Non-RCT		370	226			235	(64%)	142	(63%)		62.5	62.1		25.8	26.1		5.6	5.6			256	(69%)	160	(71%)	9
Seow-En (44)	2018	2012-2015	Non-RCT		21	6			14	(67%)	3	(50%)		NA	NA		24.0	24.0		5.0	7.0			7	(33%)	2	(33%)	9
Mege (47)	2018	2014-2017	Non-RCT	34		34	23	(68%)			23	(68%)	59.0		58.0	25.0		25.0	2.2*		1.3*	29	(85%)			29	(85%)	9
Persiani (48)	2018	2007-2017	Non-RCT	46		46	31	(67%)			30	(65%)	66.5		69.0	25.6		25.0	6.0		5.0	43	(93%)			26	(57%)	6
Roodbeen (49)	2018	2006-2017	Non-RCT	41		41	32	(78%)			34	(83%)	66.0		62.5	26.1		26.7	1.5		2.0	18	(44%)			16	(39%)	9
Rubinkiewicz (50)	2018	2012-2018	Non-RCT	35		35	24	(69%)			24	(69%)	60.3		64.3	27.1		26.1	3.2		2.9	31	(89%)			31	(89%)	8
Chen2 (45)	2019	2008-2018	Non-RCT	64		39	42	(66%)			29	(74%)	64.0		62.0	24.6		25.4	5.8		4.3	31	(48%)			15	(38%)	7
Detering (46)	2019	2015-2017	Non-RCT	396		396	281	(71%)			288	(73%)	NA		NA	NA		NA	NA		NA	156	(39%)			135	(34%)	8

*Distance to external sphincter; &Evaluated by Newcastle-Ottawa Scale; #Evaluated by Jadad score; BMI, body mass index; NA, not available.

### Definition of Mid-/Low Rectal Cancer

The most commonly used definition of mid-/low rectal cancer was the rectal adenocarcinomas with the inferior margins located within 10 cm of the anal verge. The second commonly used definition was the tumor located within 12 cm of the anal verge. For the definition of low rectal cancer, 4 studies defined low rectal cancer as a tumor located within 5 cm of the anal verge, while 2 studies defined low rectal cancer as a tumor located up to 6 cm from the anal verge. In addition to the commonly used anatomical marker of anal verge, two studies by Chouillard (36) and Velthuis (23) used the dentate line as a measuring mark to define tumor distance. Rigid proctoscopy/sigmoidoscopy was the technique most commonly used to measure the tumor distance, while Persiani et al. (48) used MRI to evaluate the distance between the distal end of the tumor and the anorectal junction (**Table 2**).

**Table 2:** Definition of mid-/low rectal cancer in included studies.

Study	Year	Definition of mid- and low rectal cancer
Bianchi (5)	2010	≤ 12 cm from the anal verge
Baek (19)	2013	NR
SY Park (20)	2013	NR
Kuo (21)	2014	NR
Denost (22)	2014	Low rectal cancer: < 6 cm from the anal verge
Velthuis (23)	2014	Low rectal cancer: 0-5 cm from the dentate line; mid rectal cancer: 5-10 cm from the dentate line
JS Park (24)	2015	Low rectal cancer: ≤ 4 cm of the anal verge
Serin (25)	2015	≤10 cm from the anal verge, measured by rigid sigmoidoscope
Yoo (26)	2015	≤ 5 cm from the anal verge, measured by digital rectal examination and/or rigid sigmoidoscopy
Chen (27)	2015	NR
De’Angelis (28)	2015	Low rectal cancer: ≤ 5 cm from the anal verge
Fernandez-Hevia (29)	2015	≤ 10 cm from the anal verge
Kanso (30)	2015	NR
Perdawood (31)	2015	≤ 10 cm from the anal verge, measured by rigid proctoscopy
Bedirli (32)	2016	Middle and lower 2/3 of rectum
Feroci (33)	2016	≤ 12 cm from the anal verge
Law (34)	2016	≤ 12 cm from the anal verge
Lim (35)	2016	≤ 12 cm from the anal verge
Chouillard (36)	2016	≤ 7 cm from the dentate line
Lelong (37)	2016	NR
Marks (38)	2016	NR
Rasulov (39)	2016	≤ 10 cm from the anal verge, measured by rigid rectoscopy
Kim (40)	2017	≤ 9 cm from the anal verge
Perez (41)	2017	Low rectal cancer: < 6cm from anal verge; mid rectal cancer: 6-12cm from anal verge
Chang (6)	2017	Low rectal cancer: ≤ 7 cm from the anal verge
Perdawood2 (42)	2017	NR
Lee (43)	2018	≤ 10 cm from the anal verge
Seow-En (44)	2018	NR
Mege (47)	2018	NR
Persiani (48)	2018	Low rectal cancer: 0–5 cm; mid rectal cancer: 5.1–10 cm, measured by MRI
Roodbeen (49)	2018	Low rectal cancer: tumor distal border was located distal to the point where the levator ani muscles insert on the pelvic bone on sagittal MRI
Rubinkiewicz (50)	2018	Low rectal cancer: <5 cm from the anal verge
Chen2 (45)	2019	≤ 7 cm from the anal verge
Detering (46)	2019	≤ 10 cm from the anal verge

### Neoadjuvant Treatment

The prevalence of patients who underwent neoadjuvant treatment varied from 16% to 100%. Concomitant radiochemotherapy was employed by the studies examined in this review, except Chen et al. (45) who did not clearly specify whether neoadjuvant chemotherapy or concomitant radiochemotherapy were adopted. Only a small number of patients received chemotherapy alone [1 patients in Roodbeen research (49) and 32 cases in Bedirli research (32)]. Induction therapy was not reported in included studies. Although the exact nature of neoadjuvant radiotherapy differed between the included studies, the majority of studies administered long-course preoperative radiochemotherapy (45 to 50.4 Gy delivered over a period of 5 to 6 weeks). Rasulov et al. (39) reported the use of long-course radiochemotherapy for T3-T4 low rectal cancer, while for other patients short-course preoperative radiochemotherapy (25 Gy) were offered. Serin et al. (25) employed short-course radiotherapy (25 Gy) for patients without risk of lateral margin positivity. Velthuis et al. (23) also reported the use of 25 Gy pelvic irradiation for low risk patients of T2-3N0-1 tumor (**Table 3**).

**Table 3:** Neoadjuvant treatment schedules in included studies.

Study	Year	Indication for neoadjuvant treatment	Neoadjuvant schedule			
			Received radiotherapy (yes/no)	Radiotherapy schedule	Received chemotherapy (yes/no)	Chemotherapy schedule
Bianchi (5)	2010	Tumor spread to the mesorectum, or N1-2 by MRI or endoscopic ultrasound	Yes	45 Gy in 5 fractions	Yes	Capecitabine
Baek (19)	2013	NR	Yes	50.4Gy, 45 Gy/25 fractions followed by a 5.4Gy boost	Yes	5-FU
SY Park (20)	2013	T3, T4, or N+	Yes	50 Gy in 25 fractions for 5 weeks	Yes	NR
Kuo (21)	2014	T3, T4, or N+	Yes	NR	Yes	NR
Denost (22)	2014	T3, T4, or N+	Yes	45 Gy in 5 weeks	Yes	5-FU and Capecitabine
Velthuis (23)	2014	T2-3N0-1 or T2-3N2	Yes	T2-3N0-1: 25 Gy in 5 fractions, T2-3N2: 50 Gy in 25 fractions	Yes	5-FU
JS Park (24)	2015	NR	Yes	50 Gy in 25 fractions for 5 weeks	Yes	NR
Serin (25)	2015	T3, T4, or N+	Yes	45–50.4 Gy; short-course radiotherapy (25 Gy pelvic irradiation) for patients without risk of lateral margin positivity	Yes	5-FU and leucoverin
Yoo (26)	2015	CRM+ or lymph nodes that escaped the TME plane	Yes	50.8 Gy in 28 fractions	Yes	5-FU based chemotherapy
Chen (27)	2015	Stage II or III	Yes	50.4Gy, 45 Gy/25 fractions followed by a 5.4Gy boost	Yes	oral 5-FU
De’Angelis (28)	2015	T3, T4N0, or T1-T4N1-N2	Yes	45–50.4 Gy delivered in daily fractions of 1.8-2 Gy for 5-6 weeks	Yes	5-FU infusion
Fernandez-Hevia (29)	2015	T3, T4N0, or T1-T4N1-N2	Yes	45 Gy/25 fractions	Yes	5-FU infusion
Kanso (30)	2015	T3, T4, or N+	Yes	50 Gy in 5 weeks	Yes	NR
Perdawood (31)	2015	T3 (≤ 5 mm from the tumor to the mesorectal fascia), T4	Yes	50.4 Gy in 28 fractions	Yes	5-FU
Bedirli (32)	2016	NR	Yes	NR	Yes, 19(54%) in Rob group and 13(46%) in Lap group only had neoadjuvant chemotherapy	NR
Feroci (33)	2016	T3, T4, or N+	Yes	NR	Yes	NR
Law (34)	2016	mesorectal margin was at risk (≤ 1 mm by MRI)	Yes	45–54 Gy	Yes	NR
Lim (35)	2016	T3, T4, or N+	Yes	50.4 Gy	Yes	5-FU
Chouillard (36)	2016	higher than T2, or N+	Yes	45-50 Gy in 5-6 weeks	Yes	5-FU
Lelong (37)	2016	T3, T4, or N+, or some T2 ultralow tumors	Yes	45-50 Gy in 25 fractions	Yes	Capecitabine
Marks (38)	2016	NR	Yes	NR	Yes	NR
Rasulov (39)	2016	mrT3abN0-1 tumors located 5-10 cm from the anal verge or T2N0-1 tumors located <5 cm from the anal verge did not receive neoadjuvant therapy	Yes	T3-T4 low rectal cancer: 50 Gy in 25 fractions; others: 25 Gy in 5 fractions	Yes	Oral capecitabine
Kim (40)	2017	T3, T4, or N+	Yes	50.4 Gy	Yes	5-FU based chemotherapy
Perez (41)	2017	NR	Yes	NR	Yes	NR
Chang (6)	2017	T0-3 N0-1	Yes	NR	Yes	NR
Perdawood2 (42)	2017	T3 (tumor at 5-10 cm from the anal verge, < 5 mm from the deepest tumor invasion to the mesorectal fascia; below 5 cm from the anal verge: all) or T4	Yes	50.8 Gy in 28 fractions	Yes	5-FU based or equivalent chemotherapy
Lee (43)	2018	T3, T4, or N+	Yes	Long course chemoradiation	Yes	NR
Seow-En (44)	2018	NR	Yes	NR	Yes	NR
Mege (47)	2018	T3, T4, or N+	Yes	50 Gy in 5 weeks	Yes	NR
Persiani (48)	2018	T3, T4, or N+	Yes	NR	Yes	NR
Roodbeen (49)	2018	NR, decided by multidisciplinary team	Yes	Long course chemoradiation	Yes, 1 patients in TaTME group received only chemotherapy	NR
Rubinkiewicz (50)	2018	T3, T4, or N+	Yes	50.4 Gy	Yes	NR
Chen2 (45)	2019	NR	NR	NR	NR	NR
Detering (46)	2019	NR	Yes	NR, including Short Course Radiotherapy-Immediate Surgery, Short Course Radiotherapy-Delayed Surgery, and Long Course Radiotherapy/Chemoradiotherapy	NR	NR

TaTME, transanal total mesorectal excision; NR, not reported.

### Primary Outcomes

**Figure 3:**
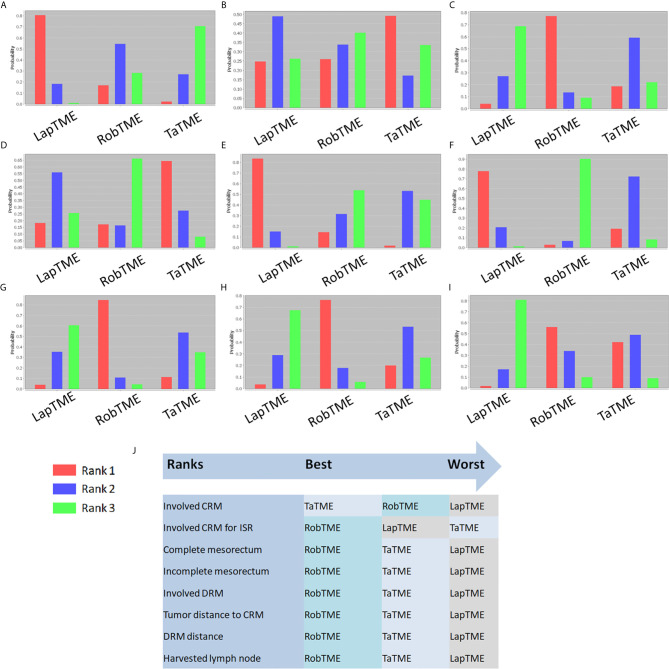
Rank probability diagram. **(A)** CRM involvement; **(B)** CRM involvement for ISR; **(C)** Complete mesorectum; **(D)** Near complete mesorectum; **(E)** Incomplete mesorectum; **(F)** DRM involvement; **(G)** Tumor distance to the CRM; **(H)** DRM distance; **(I)** Harvested lymph nodes; **(J)** Summary of surgical approach rank in terms of surgical resection quality.

The results of traditional pairwise meta-analysis and network meta-analysis are displayed in **Tables 4** and **5**, respectively. Thirty-two studies reported the rate of CRM involvement. Two of them were excluded due to different CRM definitions. Velthuis et al. (23) and Yoo et al. (26) defined CRM involvement as when the tumor was located 2 mm or less from the CRM, which might overestimate the CRM positive rate. Finally, 30 studies were included, reporting 4090 patients. Pooled data showed CRM positivity in 114 of 1763 LapTME procedures (6.5%), 54 of 1051 RobTME procedures (5.1%) and 60 of 1276 TaTME procedures (4.7%). According to the consistency test, the consistency model was used to pool the data on positive CRM rates (all the P values > 0.05 in node-splitting analysis). In addition, when the PSRFs ranged from 1.00 to 1.01, good convergence of the model was obtained. Network analysis showed that there was no significant difference among these 3 surgical approaches. The rank plot illustrating the empirical probabilities for each pathological outcome in each surgical approach ranked first through third is depicted in **Figure 3**. The transanal approach had a high probability of being the best treatment, considering that it had the lowest CRM involvement rate. The results from traditional direct pairwise meta-analysis demonstrated no significant difference regarding the CRM involvement rates between LapTME and RobTME (OR=1.312, 95% CI 0.805-2.136, P=0.275) or between LapTME and TaTME (OR=1.476, 95% CI 0.987-2.209, P=0.058). However, subgroup analysis for comparison of the positive CRM rate among types of ISR showed contrasting results. The pooled CRM involvement rate was 9.8% in the TaTME group, which was slightly higher than the rate of 9.0% in the LapTME group and 8.8% in the RobTME group, although this trend did not reach statistical significance. The RobTME group had the highest probability of being the best surgical treatment to obtain free CRMs for low rectal cancer, whereas TaTME ranked the worst.

**Table 4:** Results of traditional pair-wise meta-analysis.

Item	Comparison	I2	Model	SMD/OR (95%CI)			Z	P-value
Involved CRM	LapTME VS RobTME	0	Fixed-effect model	1.312	0.805	2.136	1.09	0.275
	LapTME VS TaTME	14.3	Fixed-effect model	1.476	0.987	2.209	1.90	0.058
Complete mesorectum	LapTME VS RobTME	21.1	Fixed-effect model	0.868	0.411	1.831	0.37	0.709
	RobTME VS TaTME	48.6	Random effects model	1.413	0.543	3.675	0.71	0.479
	LapTME VS TaTME	56.2	Random effects model	0.735	0.452	1.197	1.24	0.216
Near complete mesorectum	LapTME VS RobTME	0	Fixed-effect model	1.070	0.496	2.307	0.17	0.863
	LapTME VS TaTME	1.6	Fixed-effect model	0.806	0.573	1.132	1.24	0.214
Incomplete mesorectum	LapTME VS RobTME	54.8	Random effects model	1.612	0.065	39.911	0.29	0.770
	RobTME VS TaTME	0	Fixed-effect model	1.180	0.432	3.223	0.32	0.747
	LapTME VS TaTME	0	Fixed-effect model	1.531	0.998	2.351	1.95	0.051
Tumor distance to CRM	LapTME VS RobTME	0	Fixed-effect model	0.017	-0.179	0.213	0.17	0.863
	RobTME VS TaTME	0	Fixed-effect model	0.987	0.628	1.345	5.39	0.000
	LapTME VS TaTME	90.9	Random effects model	-0.461	-0.976	0.055	1.75	0.080
Involved DRM	LapTME VS RobTME	0	Fixed-effect model	2.268	0.415	12.389	0.95	0.344
	LapTME VS TaTME	0	Fixed-effect model	1.392	0.616	3.149	0.80	0.426
DRM distance	LapTME VS RobTME	65.8	Random effects model	-0.084	-0.279	0.111	0.84	0.398
	RobTME VS TaTME	97.1	Random effects model	0.570	-0.886	2.026	0.77	0.443
	LapTME VS TaTME	79.2	Random effects model	-0.072	-0.333	0.189	0.54	0.588
Harvested lymph node	LapTME VS RobTME	76.4	Random effects model	-0.090	-0.358	0.178	0.66	0.512
	RobTME VS TaTME	0	Fixed-effect model	0.098	-0.051	0.247	1.29	0.196
	LapTME VS TaTME	45.3	Random effects model	-0.131	-0.283	0.020	1.70	0.090

CRM, circumferential resection margin; LapTME, laparoscopic total mesorectal excision; RobTME, robotic total mesorectal excision; TaTME, transanal total mesorectal excision; ISR, intersphincteric resection; DRM, distal resection margin.

**Table 5:** Results of network meta-analysis.

	Compare with LapTME	Compare with RobTME	Compare with TaTME
Involved CRM			
LapTME		1.30 (0.71, 2.35)	1.57 (0.98, 2.73)
RobTME	0.77 (0.43, 1.41)		1.21 (0.61, 2.63)
TaTME	0.64 (0.37, 1.02)	0.83 (0.38, 1.64)	
Involved CRM for ISR			
LapTME		1.07 (0.48, 2.55)	0.86 (0.15, 3.73)
RobTME	0.94 (0.39, 2.10)		0.81 (0.12, 4.61)
TaTME	1.16 (0.27, 6.75)	1.23 (0.22, 8.21)	
Complete mesorectum			
LapTME		0.65 (0.30, 1.35)	0.86 (0.53, 1.30)
RobTME	1.53 (0.74, 3.38)		1.34 (0.60, 2.80)
TaTME	1.16 (0.77, 1.88)	0.75 (0.36, 1.67)	
Near complete mesorectum			
LapTME		1.21 (0.50, 2.78)	0.85 (0.56, 1.36)
RobTME	0.83 (0.36, 1.98)		0.70 (0.31, 1.79)
TaTME	1.17 (0.73, 1.78)	1.42 (0.56, 3.21)	
Incomplete mesorectum			
LapTME		1.99 (0.48, 11.17)	1.90 (0.99, 5.25)
RobTME	0.50 (0.09, 2.07)		0.96 (0.22, 4.43)
TaTME	0.53 (0.19, 1.01)	1.05 (0.23, 4.54)	
Involved DRM			
LapTME		8.75 (0.85, 126.50)	1.71 (0.46, 8.68)
RobTME	0.11 (0.01, 1.17)		0.20 (0.01, 1.98)
TaTME	0.58 (0.12, 2.19)	5.04 (0.50, 76.57)	
Tumor distance to CRM			
LapTME		-1.83 (-4.49, 0.71)	-0.33 (-2.17, 1.63)
RobTME	1.83 (-0.71, 4.49)		1.50 (-1.24, 4.45)
TaTME	0.33 (-1.63, 2.17)	-1.50 (-4.45, 1.24)	
DRM distance			
LapTME		-0.24 (-0.57, 0.10)	-0.08 (-0.39, 0.21)
RobTME	0.24 (-0.10, 0.57)		0.15 (-0.24, 0.54)
TaTME	0.08 (-0.21, 0.39)	-0.15 (-0.54, 0.24)	
Harvested lymph node			
LapTME		-0.96 (-2.62, 0.59)	-0.80 (-2.01, 0.49)
RobTME	0.96 (-0.59, 2.62)		0.17 (-1.59, 2.08)
TaTME	0.80 (-0.49, 2.01)	-0.17 (-2.08, 1.59)	

CRM, circumferential resection margin; LapTME, laparoscopic total mesorectal excision; RobTME, robotic total mesorectal excision; TaTME, transanal total mesorectal excision; ISR, intersphincteric resection; DRM, distal resection margin.

### Secondary Outcomes

Nineteen studies reported the mesorectal quality. The consistency test showed good consistency between direct comparisons and indirect estimated comparisons (all the P values > 0.05 in node-splitting analysis), and good convergence of the model was obtained (all PSRFs ranged from 1.00 to 1.01) for all mesorectal quality outcomes. Complete mesorectal excision was observed in 541 (75.1%) of 720 patients who underwent LapTME, in 547 (93.7%) of 584 patients who underwent RobTME and in 647 (81.2%) of 797 patients who underwent TaTME. The network analysis results showed no significant difference in the complete mesorectal excision rates among these 3 surgical approaches. The results from traditional direct pairwise meta-analysis demonstrated no significant difference for complete mesorectal excision between LapTME and RobTME (OR=0.868, 95% CI 0.411-1.831, P=0.709), between RobTME and TaTME (OR=1.413, 95% CI 0.543-3.675, P=0.479), or between LapTME and TaTME (OR=0.735, 95% CI 0.452-1.197, P=0.216). In addition, RobTME ranked best with the highest probability for complete mesorectal excision.

The incomplete mesorectal excision rates were 9.6% (69/720) in the LapTME group, 1.9% (11/584) in the RobTME group and 5.6% (45/797) in the TaTME group. Pooled network analysis observed a higher but not statistically significant risk of incomplete mesorectum when comparing both LapTME with RobTME (OR = 1.99; 95% CI = 0.48-11.17) and LapTME with TaTME (OR = 1.90; 95% CI = 0.99-5.25). According to the results of direct pairwise meta-analysis, there was no significant difference between LapTME and RobTME (OR=1.612, 95% CI 0.065-39.911, P=0.770), RobTME and TaTME (OR=1.180, 95% CI 0.432-3.223, P=0.747), or LapTME and TaTME (OR=1.531, 95% CI 0.998-2.351, P=0.051). Moreover, LapTME ranked the worst for obtaining incomplete mesorectal excision. Compared with TaTME, RobTME achieved a greater mean tumor distance to the CRM (WMD, 0.987; 95% CI 0.628–1.345; P < 0.001) in both the direct comparison and indirect network estimated comparison (WMD, 4.31; 95% CI 0.38–7.78). The inconsistency model was used because all the P values < 0.05 in node-splitting analysis.

Sixteen studies reported DRMs. The pooled DRM positivity rate was 2.0% (14/706) for the LapTME group, 0.3% (2/739) for the RobTME group and 1.9% (12/638) for the TaTME group. Synthesis of the results found that the DRM positive rate was not affected by the 3 different approaches. Similarly, no differences in the length of DRMs among the 3 surgical approaches were found. No significant difference was discovered between the two groups with respect to the length of DRMs (LapTME *vs* RobTME, WMD = -0.084, 95% CI -0.279-0.111, P = 0.398; RobTME vs TaTME, WMD = 0.570, 95% CI -0.886-2.026, P = 0.443; LapTME vs TaTME, WMD = -0.072, 95% CI -0.333-0.189, P = 0.588). RobTME ranked the best with a high probability for the lowest rate of DRM involvement, the longest length of DRMs, and the number of harvested lymph nodes.

## Discussion

Since TME for mid-/low rectal cancer has been elucidated to optimize locoregional clearance (51), the LR rate has decreased to approximately 6% (52), and an optimal surgical approach for mid-/low rectal cancer has yet to be achieved. The quality of surgical resection metrics for rectal cancer is defined and evaluated by positive CRMs, incomplete planes of mesorectal excision and positive DRMs (53). The relationship between the quality of surgical resection and long-term oncological outcomes has been well established (53), and the quality of surgical resection has been recommended for evaluating novel surgical interventions (54). Compared with previous reports of meta-analyses (7, 8), this study used network meta-analysis to comprehensively estimate the quality of surgical resection in RobTME, TaTME, and LapTME for mid-/low rectal cancer treatment. The results demonstrated that RobTME achieved a greater mean tumor distance to the CRM than TaTME. In addition, no difference was observed in terms of the CRM involvement rates and all other surgical resection quality variables among RobTME, TaTME, and LapTME. By comparison, RobTME was most likely to be ranked the best in terms of CRM involvement, complete mesorectal excision, rate of DRM involvement and length of DRMs. TaTME was most likely to be ranked the worst in terms of CRM involvement for ISR in low rectal cancer.

The CRM was introduced as a powerful prognostic factor for rectal cancer resection and an important index for measuring the curative effect of surgery. Many large-scale studies have been published demonstrating the value of CRM involvement for local recurrence, overall recurrence and cancer-specific mortality (55, 56). Moreover, since cancer metastases have been found to spread to the distal mesorectum in approximately 40% of rectal cancer cases, a potentially residual disease in the distal mesorectum predisposes patients to pelvic recurrence (57). However, tapering of the distal mesorectum makes radical resection of mid- and low rectal lesions difficult with laparoscopy. Previous RCTs found that CRM rates varied from 4.0% to 15.5% with the laparoscopic approach (58, 59), in line with the results of our pooled CRM rate of 6.5%. However, a higher CRM rate of 9.0% in laparoscopic ISR was found in the present study, coinciding with a rate of 9.4% in a previous multicenter study (24). For this reason, TaTME has been developed as an alternative technique for the treatment of mid-/low rectal cancer, as TaTME provides better dissection of the presacral plane and the rectoprostatic plane or rectovaginal plane with better visualization of the distal rectum (60). Although TaTME performed in mid-/low rectal cancer patients has shown encouraging results (61), its oncological feasibility and safety are yet to be verified through ongoing large RCTs (COLOR III), as the results are only expected approximately in the year 2022 (62). Prior traditional systematic reviews by Hu et al. (63) and Wu et al. (64) comparing LapTME to TaTME for mid-/low rectal cancer showed that TaTME was associated with a reduced positive CRM rate and could achieve complete tumor resection with improved long-term survival. Rubinkiewicz et al. (65) conducted an updated meta-analysis to compare the pure standard LapTME and TaTME procedures by excluding studies on abdominoperineal resection or cases of Hartmann resection. No significant differences regarding the CRM, completeness of mesorectal excision, or DRM were found. However, the sample size in the TaTME group in these previous meta-analyses (414 cases in the Hu study, 348 cases in the Wu study, and 358 in the Rubinkiewicz study) was still insufficient, which could have influenced the statistical significance. Investigators of the COLOR III study estimated that at least 732 patients would be required for the TaTME arm to demonstrate a CRM difference based on an estimated CRM rate of 7% (62). The estimated CRM rate in the COLOR III study was quite similar to our current pooled results. However, with a total of 1362 patients included in the TaTME group of the present network meta-analysis, we failed to find a benefit of TaTME in terms of the CRM rate. Admittedly, the surgical techniques used in TaTME might not be standardized, and performing TME from below is challenging due to the limited anatomical landmarks.

A robotic-assisted approach, another alternative technique, provides wrist motion for endoscopic instruments to overcome several of the technical difficulties associated with laparoscopy. A lower rate of CRM involvement (OR = 0.5) was found to be associated with RobTME in an early meta-analysis containing 592 patients (324 in the RobTME group and 268 in the LapTME group) (66). However, the early results of a recent RCT (ROLARR trial) showed that CRM involvement rates were comparable between robotic-assisted (5.1%) and conventional laparoscopic (6.3%) rectal cancer resection, in accordance with an updated systematic review following the publication of the ROLARR trial (67) and our current results.

Although little data exist regarding head-to-head comparative analyses of RobTME and TaTME, our Bayesian network meta-analysis allowed us to compare these 3 techniques indirectly and gain more precise effect estimates by collectively evaluating direct and indirect comparisons. Although no difference was observed in terms of CRM involvement among these 3 techniques, RobTME achieved a significantly safer CRM rate than TaTME. In addition, the present study showed that RobTME had a high probability of being the best surgical treatment with regard to CRM involvement in ISR procedures. This was probably due to the ability of the versatile instruments to dissect as far caudally as the intersphincteric space while compensating for the challenges posed by the deep pelvis. Two published network meta-analyses (7, 8) comparing these 3 surgical techniques in rectal cancer came to different conclusions about CRM involvement because of several substantial biases. First, the network meta-analysis by Simillis et al. (7) had seriously different sample sizes among the 4 different surgical techniques compared. Only 50 TaTME cases were included compared to 2350 open, 3276 laparoscopic, and 561 robotic cases, which resulted in very large statistical errors. For instance, the odds ratio for the comparison of conversion rates between TaTME and open TME was as high as 4964, with a large 95% CI (0.6- 39,611,894). Second, it might be considered that all the TaTME studies included in the two previous network meta-analyses included only patients with mid-/low rectal cancers, unlike data from LapTME and RobTME studies that also included upper rectal cancers. CRM involvement rates were lower in operations for upper rectal cancer than for low rectal cancer operations in previous studies (68, 69). Furthermore, partial mesorectal excision (PME) with mesorectal transection 5 cm below the tumor is adequate for upper rectal cancers, while TME is necessary for mid-/low rectal cancer. This might decrease the diversity and strength of a network meta-analysis when performing indirect comparisons and calculating treatment rankings with probabilities among LapTME, RobTME, and TaTME. Last but not least, since the cutoff value for defining CRM positivity is still under debate, the threshold of 1 mm or less is the most commonly accepted and used in included studies (70). Rausa (8), in their network meta-analysis, also included the study by Velthuis, in which a positive CRM was defined as tumor involvement of 2 mm or less from the resection margin. The pooled result was therefore questionable due to a combination of CRM involvement rates with inconsistent definitions. In the present study, we included only mid-/low rectal cancers and defined CRM positivity with a threshold of 1 mm or less. Moreover, the sample sizes were relatively comparable among the different surgical approach groups.

Compared with the CRM, the mesorectal quality, or the so-called plane of mesorectal excision, represented a stricter and more precise indicator for assessing the degree of radical surgical resection. Since CRM involvement might occur when cT4 tumors grow directly into the circumferential margin, this cannot be considered incomplete removal of the surrounding mesorectum. Incomplete mesorectal excision might not always be related to CRM involvement in the case of a small tumor. In our study, there were no differences between these 3 surgical approaches regarding the quality of the specimen. RobTME ranked the best with a high probability of complete mesorectal excision. Furthermore, pooled network analysis observed a higher but not statistically significant risk of incomplete mesorectum when comparing both LapTME with RobTME (OR = 1.99) and LapTME with TaTME (OR = 1.90). However, we believe the results of the present study should be carefully interpreted. The incomplete mesorectal excision rate was obviously higher in the LapTME group (9.6%) than the rate of 1.9% in the RobTME group and of 5.6% in the TaTME group, even though these differences did not reach statistical significance. Additionally, a study based on postoperative magnetic resonance imaging (MRI) of the pelvis found that residual mesorectal tissue was detected in 3.1% of TaTME patients and 46.9% of LapTME patients, which indicated that the completeness of mesorectal excision was significantly better with TaTME than with the standard laparoscopic technique (71). Since the association of TME quality with prognosis was established in a previous large-scale RCT (72), the oncological outcomes of direct comparison among these 3 techniques when a negative CRM is combined with intact mesorectal excision should be awaited to specifically assess the surgical quality.

In laparoscopic surgery, it is challenging to accurately identify the distal margin and apply endoscopic staplers at a right angle to the rectum within the limited dissecting space. In TaTME, the tumor is distally approached through the anus, and the use of linear staplers can be avoided. This facilitates surgeons to accurately determine the DRM and logically secure a safe, adequate DRM length before rectal transection. Interestingly, however, we found a similar DRM positivity rate between LapTME (2.0%) and TaTME (1.9%), both of which were higher than the rate of RobTME (0.3%), even though the differences did not reach statistical significance. This could be explained by DRM involvement due to residual tumor cells beyond the regressed tumor edge after neoadjuvant chemoradiation. Surgeons might perform frozen sections to ensure oncologic clearance during TaTME for advanced tumors that have been subjected to neoadjuvant therapy. However, there is insufficient evidence in the literature regarding this issue. Moreover, it should be emphasized that the heterogeneity of the height of tumors from the anal verge and the proportion of neoadjuvant therapy might cause bias. In addition, RobTME was ranked the best with a high probability for the lowest rate of DRM involvement and longest DRM length in this study. These results are comparable to those of a recently published multicenter matched study (43) and a previous retrospective study (33). The potential advantage of RobTME regarding DRMs may be the result of technical advantages of the robotic approach because it allows the surgeon to perform high-quality maneuvers in the narrow pelvic cavity.

It is worth noting that TaTME was most likely to be ranked the worst in terms of CRM involvement for ISR in low rectal cancer. Larsen et al. (73) reported a 9.5% local recurrence rate at a median follow-up of 11 months among Norwegian rectal cancer patients who underwent TaTME. This rate is twice that of the rate observed in the COLOR II study (74), which included laparoscopic and open surgery cases, and has prompted the nationwide cessation of TaTME. In addition, these cases of local recurrence occurred early and with multifocal pelvic sidewall involvement. One explanation is that the rate of CRM involvement in TaTME for rectal cancers from this Norwegian national cohort was 12.7% (75), which was higher than the rates in RobTME and LapTME (67), consistent with our findings. The other explanation is due to the disadvantages of transanal dissection related to rectal transection and air flow during dissection from the perineum.

There were some limitations in our present study. First, except for 2 RCTs, the other studies included were all retrospective comparative studies, which created bias in the selection of patients for each minimally invasive procedure, especially during the learning curve period. However, coarsened exact matching was conducted by Lee, and propensity score matching analysis was performed by Persiani and Detering to eliminate selection bias. Moreover, our primary outcome of interest, the CRM, was routinely collected and objectively measured, thereby minimizing the problems of reporting bias due to the retrospective nature of the analysis. Second, the tumor response to neoadjuvant radiotherapy was demonstrated to have affected margin involvement (76). Although the CRM involvement rate was similar among these 3 approaches, the incomplete mesorectal excision rate was obviously higher in the LapTME group (9.6%) than the rate of 1.9% in the RobTME group and of 5.6% in the TaTME group, without a significant difference. Further RCTs stratified on the basis of neoadjuvant treatment are needed to specifically assess the surgical qualities of these 3 approaches. Third, although margin status and mesorectal excision completeness are important variables to assess resection quality, oncological outcomes are a multifactorial phenomenon. Long-term follow-up is awaited to assess the oncological outcomes among each minimally invasive procedure.

Based on the available data pooled from the most recent evidence, no difference was observed in terms of CRM involvement rates and all other qualities of surgical resection variables among RobTME, TaTME, and LapTME. Overall, RobTME was most likely to be ranked the best in terms of the quality of surgical resection for the treatment of mid-/low rectal cancer. In addition, TaTME should be performed with caution in the treatment of low rectal cancer.

## Data Availability Statement

The original contributions presented in the study are included in the article/supplementary material. Further inquiries can be directed to the corresponding authors.

## Author Contributions

XW, ZZ, QY, SL, and PC conceived and designed the study. XW, QY, WG, XL, YH, and SH contributed to the computational analyses and confirmed the results. All authors contributed to the article and approved the submitted version.

## Funding

This study was financially supported by the National Clinical Key Specialty Construction Project (General Surgery) of China (No. 2012-649), National Natural Science Foundation of China(81902378), Young Scientist Foundation of Fujian Provincial Commission of Health and Family Planning (2017-1-39) and the Startup Fund for Scientific Research, Fujian Medical University (No. 2017XQ1028), Joint Funds for the innovation of science and Technology, Fujian province (2017Y9038; 2017Y9103), Fujian Science and Technology Project (2016J01456), Natural Science Foundation of Fujian Province (2020J011030), Medical Science Research Foundation of Beijing Medical and Health Foundation (B20062DS), Fujian provincial health technology project (2020CXA025), Bethune Charitable Foundation (X-J-2018-004).

## Conflict of Interest

The authors declare that the research was conducted in the absence of any commercial or financial relationships that could be construed as a potential conflict of interest.

## Publisher’s Note

All claims expressed in this article are solely those of the authors and do not necessarily represent those of their affiliated organizations, or those of the publisher, the editors and the reviewers. Any product that may be evaluated in this article, or claim that may be made by its manufacturer, is not guaranteed or endorsed by the publisher.

## References

[1] Martling A Singnomklao T Holm T Rutqvist LE Cedermark B. Prognostic Significance of Both Surgical and Pathological Assessment of Curative Resection for Rectal Cancer. Br J Surg (2010) 91(8):1040–5. 10.1002/bjs.4557 15286968

[2] Wang CL Qu G Xu HW. The Short- and Long-Term Outcomes of Laparoscopic Versus Open Surgery for Colorectal Cancer: A Meta-Analysis. Int J Colorectal Dis (2014) 29(3):309–20. 10.1007/s00384-013-1827-1 24445673

[3] Stevenson ARL Solomon MJ Lumley JW Peter H Clouston AD Gebski VJ. Effect of Laparoscopic-Assisted Resection vs Open Resection on Pathological Outcomes in Rectal Cancer: The ALaCaRT Randomized Clinical Trial. JAMA (2015) 314(13):1356–63. 10.1001/jama.2015.12009 26441180

[4] James F Megan B Sargent DJ Anne Marie B Virgilio G Maher A. Effect of Laparoscopic-Assisted Resection vs Open Resection of Stage II or III Rectal Cancer on Pathologic Outcomes: The ACOSOG Z6051 Randomized Clinical Trial. JAMA (2015) 314(13):1346–55. 10.1001/jama.2015.10529 PMC5140087 26441179

[5] Bianchi PP Ceriani C Locatelli A Spinoglio G Zampino MG Sonzogni A. Robotic Versus Laparoscopic Total Mesorectal Excision for Rectal Cancer: A Comparative Analysis of Oncological Safety and Short-Term Outcomes. Surg Endoscopy Other Interventional Techniques (2010) 24(11):2888–94. 10.1007/s00464-010-1134-7 20526623

[6] Chang TC Kiu KT. Transanal Total Mesorectal Excision in Lower Rectal Cancer: Comparison of Short-Term Outcomes With Conventional Laparoscopic Total Mesorectal Excision. J Laparoendoscopic Adv Surg Tech A (2018) 28:365–9. 10.1089/lap.2017.0520 29190178

[7] Simillis C Lal N Thoukididou SN Kontovounisios C Smith JJ Hompes R. Open Versus Laparoscopic Versus Robotic Versus Transanal Mesorectal Excision for Rectal Cancer: A Systematic Review and Network Meta-Analysis. Ann Surg (2019) 270:59–68. 10.1097/SLA.0000000000003227 30720507

[8] Rausa E Bianco F Kelly ME Aiolfi A Petrelli F Bonitta G. Systemic Review and Network Meta-Analysis Comparing Minimal Surgical Techniques for Rectal Cancer: Quality of Total Mesorectum Excision, Pathological, Surgical, and Oncological Outcomes. J Surg Oncol (2019) 119:987–98. 10.1002/jso.25410 30811043

[9] Nagtegaal ID van de Velde CJ van der Worp E Kapiteijn E Quirke P van Krieken JH. Cooperative Clinical Investigators of the Dutch Colorectal Cancer Group. Macroscopic Evaluation of Rectal Cancer Resection Specimen: Clinical Significance of the Pathologist in Quality Control. J Clin Oncol (2002) 20(7):1729–34. 10.1200/JCO.2002.07.010 11919228

[10] Heald RJ Ryall RD. Recurrence and Survival After Total Mesorectal Excision for Rectal Cancer. Lancet (1986) 327(8496):1479–82. 10.1016/S0140-6736(86)91510-2 2425199

[11] Moher D Liberati A Tetzlaff J Altman DG. Preferred Reporting Items for Systematic Reviews and Meta-Analyses: The PRISMA Statement. *PLoS Med* (2009) 6:e1000097. 10.1371/journal.pmed.1000097 PMC2707599 19621072

[12] Jadad AR Moore RA Carroll D Jenkinson C Reynolds DJ Gavaghan DJ. Assessing the Quality of Reports of Randomized Clinical Trials: Is Blinding Necessary? Control Clin Trials (1996) 17:1–12. 10.1016/0197-2456(95)00134-4 8721797

[13] Wells GA Shea BJ O’Connell D Peterson J Welch V Losos M. The Newcastle-Ottawa Scale (NOS) for Assessing the Quality of Non-Randomised Studies in Meta-Analyses. Ottawa: Department of Epidemiology and Community Medicine, University of Ottawa (2005). Available at: http://www.ohri.ca/programs/clinical_epidemiology/oxford.asp.

[14] Ri HM Won PJ Sohee P Hyekyoung Y Dae Yong K Hee Jin C. Prognostic Impact of Circumferential Resection Margin in Rectal Cancer Treated With Preoperative Chemoradiotherapy. Ann Surg Oncol (2014) 21(4):1345–51. 10.1245/s10434-014-3484-1 24468928

[15] Hozo SP Djulbegovic B Hozo I. Estimating the Mean and Variance From the Median, Range, and the Size of a Sample. BMC Med Res Method (2005) 5(1):13. 10.1186/1471-2288-5-13 PMC1097734 15840177

[16] Brooks SP Gelman A. General Methods for Monitoring Convergence of Iterative Simulations. J Comput Graphical Stat (1998) 7(4):434–55. 10.1080/10618600.1998.10474787

[17] Higgins JPT Thompson SG Deeks JJ Altman DG. Measuring Inconsistency in Meta-Analyses. BMJ (2003) 327:557–60. 10.1136/bmj.327.7414.557 PMC192859 12958120

[18] Dias S Welton NJ Caldwell DM Ades AE. Checking Consistency in Mixed Treatment Comparison Meta-Analysis. Stat Med (2010) 29(7–8):932–44. 10.1002/sim.3767 20213715

[19] Se Jin B Sami AA Duck Hyoun J Hyuk H Byung Soh M Seung Hyuk B. Robotic Versus Laparoscopic Coloanal Anastomosis With or Without Intersphincteric Resection for Rectal Cancer. Surg Endoscopy (2013) 27(11):4157–63. 10.1007/s00464-013-3014-4 23708725

[20] Park SY Choi GS Park JS Kim HJ Ryuk JP. Short-Term Clinical Outcome of Robot-Assisted Intersphincteric Resection for Low Rectal Cancer: A Retrospective Comparison With Conventional Laparoscopy. Surg Endoscopy (2013) 27(1):48. 10.1007/s00464-012-2405-2 22752275

[21] Kuo LJ Lin YK Chang CC Tai CJ Chiou JF Chang YJ. Clinical Outcomes of Robot-Assisted Intersphincteric Resection for Low Rectal Cancer: Comparison With Conventional Laparoscopy and Multifactorial Analysis of the Learning Curve for Robotic Surgery. Int J Colorectal Dis (2014) 29(5):555–62. 10.1007/s00384-014-1841-y 24562546

[22] Quentin D Jean-Philippe A Anne R Etienne B Christophe L Eric R. Perineal Transanal Approach: A New Standard for Laparoscopic Sphincter-Saving Resection in Low Rectal Cancer, a Randomized Trial. Ann Surg (2014) 260(6):993–9. 10.1097/SLA.0000000000000766 24950270

[23] Velthuis S Nieuwenhuis DH Ruijter TEG Cuesta MA Bonjer HJ Sietses C. Transanal Versus Traditional Laparoscopic Total Mesorectal Excision for Rectal Carcinoma. Surg Endoscopy (2014) 28(12):3494–9. 10.1007/s00464-014-3636-1 24972923

[24] Park JS Kim NK Kim SH Lee KY Lee KY Shin JY. Multicentre Study of Robotic Intersphincteric Resection for Low Rectal Cancer. Br J Surg (2015) 102(12):1567–73. 10.1002/bjs.9914 26312601

[25] Serin KR Gultekin FA Batman B Ay S Kapran Y Saglam S. Robotic Versus Laparoscopic Surgery for Mid or Low Rectal Cancer in Male Patients After Neoadjuvant Chemoradiation Therapy: Comparison of Short-Term Outcomes. J Robotic Surg (2015) 9(3):187. 10.1007/s11701-015-0514-3 26531198

[26] Yoo BE Cho JS Shin JW Lee DW Kwak JM Jin K. Robotic Versus Laparoscopic Intersphincteric Resection for Low Rectal Cancer: Comparison of the Operative, Oncological, and Functional Outcomes. Ann Surg Oncol (2015) 22(4):1219–25. 10.1245/s10434-014-4177-5 25326398

[27] Chen CC Lai YL Jiang JK Chu CH Huang IP Chen WS. Transanal Total Mesorectal Excision Versus Laparoscopic Surgery for Rectal Cancer Receiving Neoadjuvant Chemoradiation: A Matched Case–Control Study. Ann Surg Oncol (2015) 23(4):1–8. 10.1245/s10434-015-4997-y 26597369

[28] De’Angelis N Portigliotti L Azoulay D Brunetti F. Transanal Total Mesorectal Excision for Rectal Cancer: A Single Center Experience and Systematic Review of the Literature. Langenbecks Arch Surg (2015) 400(8):945–59. 10.1007/s00423-015-1350-7 26497544

[29] María FH Salvadora D Antoni C Marta T Dulce M Gabriel DDG. Transanal Total Mesorectal Excision in Rectal Cancer: Short-Term Outcomes in Comparison With Laparoscopic Surgery. Ann Surg (2015) 261(2):221. 10.1097/SLA.0000000000000865 25185463

[30] Kanso F Maggiori L Debove C Chau A Ferron M Panis Y. Perineal or Abdominal Approach First During Intersphincteric Resection for Low Rectal Cancer: Which Is the Best Strategy? Dis Colon Rectum (2015) 58(7):637. 10.1097/DCR.0000000000000396 26200677

[31] Perdawood SK Al Khefagie GA. Transanal vs Laparoscopic Total Mesorectal Excision for Rectal Cancer: Initial Experience From Denmark. Colorectal Dis (2016) 18(1):51–8. 10.1111/codi.13225 26603786

[32] Bedirli A Salman B Yuksel O. Robotic Versus Laparoscopic Resection for Mid and Low Rectal Cancers. Jsls J Soc Laparoendoscopic Surgeons (2016) 20(1):e2015.00110. 10.4293/JSLS.2015.00110 PMC4824844 27081292

[33] Feroci F Vannucchi A Bianchi PP Cantafio S Garzi A Formisano G. Total Mesorectal Excision for Mid and Low Rectal Cancer: Laparoscopic vs Robotic Surgery. World J Gastroenterol (2016) 22(13):3602–10. 10.3748/wjg.v22.i13.3602 PMC4814646 27053852

[34] Law WL Foo DC. Comparison of Short-Term and Oncologic Outcomes of Robotic and Laparoscopic Resection for Mid-and Distal Rectal Cancer. Surg Endoscopy (2017) 31(7):2798–807. 10.1007/s00464-016-5289-8 27785627

[35] Lim DR Bae SU Hur H Min BS Baik SH Kang YL. Long-Term Oncological Outcomes of Robotic Versus Laparoscopic Total Mesorectal Excision of Mid–Low Rectal Cancer Following Neoadjuvant Chemoradiation Therapy. Surg Endoscopy Other Interventional Techniques (2016) 31(4):1–10. 10.1007/s00464-016-5165-6 27631313

[36] Canonico G D''Alessandro A Chouillard E. Transanal Notes Total Mesorectal Excision (TME) in Patients With Rectal Cancer: Is Anatomy Better Preserved? Techniques Coloproctol (2016) 44(4):1–8. 10.1016/j.ejso.2018.01.060 26993638

[37] Lelong B Meillat H Zemmour C Poizat F Ewald J Mege D. Short-And Mid-Term Outcomes After Endoscopic Transanal or Laparoscopic Transabdominal Total Mesorectal Excision for Low Rectal Cancer: A Single Institutional Case-Control Study. J Am Coll Surgeons (2017) 224(5):917–25. 10.1016/j.jamcollsurg.2016.12.019 28024946

[38] Marks JH Montenegro GA Salem JF Shields MV Marks GJ. Transanal TATA/TME: A Case-Matched Study of taTME Versus Laparoscopic TME Surgery for Rectal Cancer. Techniques Coloproctol (2016) 20(7):467–73. 10.1007/s10151-016-1482-y 27178183

[39] Rasulov AO Mamedli ZZ Gordeyev SS Kozlov NA Dzhumabaev HE. Short-Term Outcomes After Transanal and Laparoscopic Total Mesorectal Excision for Rectal Cancer. Techniques Coloproctol (2016) 20(4):227. 10.1007/s10151-015-1421-3 26794213

[40] Kim MJ Park SC Park JW Chang HJ Kim DY Nam BH. Robot-Assisted Versus Laparoscopic Surgery for Rectal Cancer: A Phase II Open Label Prospective Randomized Controlled Trial. Ann Surg (2017) 267(2):243. 10.1097/SLA.0000000000002321 28549014

[41] Perez D Melling N Biebl M Reeh M Baukloh JK Miro J. Robotic Low Anterior Resection Versus Transanal Total Mesorectal Excision in Rectal Cancer: A Comparison of 115 Cases. Eur J Surg Oncol (2018) 44:237–42. 10.1016/j.ejso.2017.11.011 29249592

[42] Perdawood SK Thinggaard BS Bjoern MX. Effect of Transanal Total Mesorectal Excision for Rectal Cancer: Comparison of Short-Term Outcomes With Laparoscopic and Open Surgeries. Surg Endoscopy (2018) 32:2312–21. 10.1007/s00464-017-5926-x 29098433

[43] Lee L Lacy BD Ruiz MG Liberman AS Albert MR Monson JRT. A Multicenter Matched Comparison of Transanal and Robotic Total Mesorectal Excision for Mid and Low-Rectal Adenocarcinoma. Ann Surg (2019) 270:1110–6. 10.1097/SLA.0000000000002862 29916871

[44] Seow-En I Seow-Choen F. An Initial Experience Comparing Robotic Total Mesorectal Excision (RTME) and Transanal Total Mesorectal Excision (taTME) for Low Rectal Tumours. Ann Acad Med Singapore (2018) 47(5):188–90. 29911735

[45] Chen Y-T Kiu K-T Yen M-H Chang T-C. Comparison of the Short-Term Outcomes in Lower Rectal Cancer Using Three Different Surgical Techniques: Transanal Total Mesorectal Excision (TME), Laparoscopic TME, and Open TME. Asian J Surg (2019) 42(6):674–80. 10.1016/j.asjsur.2018.09.008 30318319

[46] Detering R Roodbeen SX van Oostendorp SE Dekker J-WT Sietses C Bemelman WA. Three-Year Nationwide Experience With Transanal Total Mesorectal Excision for Rectal Cancer in the Netherlands: A Propensity Score Matched Comparison With Conventional Laparoscopic Total Mesorectal Excision. J Am Coll Surgeons (2019) 228(3):235–44.e1. 10.1016/j.jamcollsurg.2018.12.016 30639298

[47] Mege D Hain E Lakkis Z Maggiori L Prost à la Denise J Panis Y. Is Trans-Anal Total Mesorectal Excision Really Safe and Better Than Laparoscopic Total Mesorectal Excision With a Perineal Approach First in Patients With Low Rectal Cancer? A Learning Curve With Case-Matched Study in 68 Patients. Colorectal Dis (2018) 20(6):O143–51. 10.1111/codi.14238 29693307

[48] Persiani R Biondi A Pennestrì F Fico V D’Ugo D. Transanal Total Mesorectal Excision vs Laparoscopic Total Mesorectal Excision in the Treatment of Low and Middle Rectal Cancer: A Propensity Score Matching Analysis. Dis Colon Rectum (2018) 61(7):1. 10.1097/DCR.0000000000001063 29771810

[49] Roodbeen SX Penna M Mackenzie H Kusters M Slater A Jones OM. Transanal Total Mesorectal Excision (TaTME) Versus Laparoscopic TME for MRI-Defined Low Rectal Cancer: A Propensity Score-Matched Analysis of Oncological Outcomes. Surg Endosc (2019) 33:2459–67. 10.1007/s00464-018-6530-4 PMC6647375 30350103

[50] Rubinkiewicz M Nowakowski M Wierdak M Mizera M Dembiński M Pisarska M. Transanal Total Mesorectal Excision for Low Rectal Cancer: A Case-Matched Study Comparing TaTMe Versus Standard Laparoscopic TMe. Cancer Manage Res (2018) 10:5239. 10.2147/CMAR.S181214 PMC6219401 30464621

[51] Heald RJ Husband EM Ryall RD. The Mesorectum in Rectal Cancer Surgery—the Clue to Pelvic Recurrence? Br J Surg (1982) 69:613–6. 10.1002/bjs.1800691019 6751457

[52] Leroy J Jamali F Forbes L Smith M Rubino F Mutter D. Laparoscopic Total Mesorectal Excision (TME) for Rectal Cancer Surgery: Long-Term Outcomes. Surg Endoscopy Other Interventional Techniques (2004) 18(2):281–9. 10.1007/s00464-002-8877-8 14691716

[53] Acuna SA Chesney TR Amarasekera ST Baxter NN. Defining Non-Inferiority Margins for Quality of Surgical Resection for Rectal Cancer: A Delphi Consensus Study. Ann Surg Oncol (2018) 25(11):3171–8. 10.1245/s10434-018-6639-7 30051366

[54] Acuna S Ramjist J Baxter N Chesney T. Laparoscopic Versus Open Resection for Rectal Cancer: A Non-Inferiority Meta-Analysis of Quality of Surgical Resection Outcomes. Ann Surg (2019) 269:849–55. 10.1097/SLA.0000000000003072 30339624

[55] Liu Q Luo D Cai S Li Q Li X. Circumferential Resection Margin as a Prognostic Factor After Rectal Cancer Surgery: A Large Population-Based Retrospective Study. Cancer Med (2018) 7:3673–81. 10.1002/cam4.1662 PMC6089167 29992773

[56] Shin DW Shin JY Oh SJ Park JK Yu H Ahn MS. The Prognostic Value of Circumferential Resection Margin Involvement in Patients With Extraperitoneal Rectal Cancer. Am Surgeon (2016) 82(4):348. 10.1177/000313481608200421 27097629

[57] Wexner SD. Total Mesorectal Excision and Low Rectal Anastomosis for the Treatment of Rectal Cancer and Prevention of Pelvic Recurrences. Techniques Coloproctol (2001) 5(3):216–20. 10.1001/archsurg.136.2.216 11892031

[58] van der Pas MH Haglind E Cuesta MA Fürst A Lacy AM Hop WC. Laparoscopic Versus Open Surgery for Rectal Cancer (COLOR II): Short-Term Outcomes of a Randomised, Phase 3 Trial. Lancet Oncol (2013) 14(3):210–8. van der Pas. 10.1016/S1470-2045(13)70016-0 23395398

[59] Guillou PJ Quirke P Thorpe H Walker J Jayne DG Smith AM. Short-Term Endpoints of Conventional Versus Laparoscopic-Assisted Surgery in Patients With Colorectal Cancer (MRC CLASICC Trial): Multicentre, Randomised Controlled Trial. Lancet (2005) 365(9472):1718–26. 10.1016/S0140-6736(05)66545-2 15894098

[60] Vignali A Elmore U Milone M Rosati R. Transanal Total Mesorectal Excision (TaTME): Current Status and Future Perspectives. Updates Surg (2019) 71:29–37. 10.1007/s13304-019-00630-7 30734896

[61] Lacy AM Tasende MM Delgado S Fernandez-Hevia M Jimenez M Lacy BD. Transanal Total Mesorectal Excision for Rectal Cancer: Outcomes After 140 Patients. J Am Coll Surgeons (2015) 221(2):415–23. 10.1016/j.jamcollsurg.2015.03.046 26206640

[62] Deijen CL Velthuis S Tsai A Mavroveli S Klerk LD Sietses C. COLOR III: A Multicentre Randomised Clinical Trial Comparing Transanal TME Versus Laparoscopic TME for Mid and Low Rectal Cancer. Surg Endoscopy Other Interventional Techniques (2016) 30(8):3210–5. 10.1007/s00464-015-4615-x PMC4956704 26537907

[63] Hu D Jin P Hu L Liu W Zhang W Guo T. The Application of Transanal Total Mesorectal Excision for Patients With Middle and Low Rectal Cancer: A Systematic Review and Meta-Analysis. Medicine (2018) 97(28):e11410. 10.1097/MD.0000000000011410 29995787 PMC6076192

[64] Wu Z Zhou W Chen F Wang W Feng Y. Short-Term Outcomes of Transanal Versus Laparoscopic Total Mesorectal Excision: A Systematic Review and Meta-Analysis of Cohort Studies. J Cancer (2019) 10(2):341. 10.7150/jca.27830 30719128 PMC6360303

[65] Rubinkiewicz M Czerwińska A Zarzycki P Małczak P Nowakowski M Major P. Comparison of Short-Term Clinical and Pathological Outcomes After Transanal Versus Laparoscopic Total Mesorectal Excision for Low Anterior Rectal Resection Due to Rectal Cancer: A Systematic Review With Meta-Analysis. J Clin Med (2018) 7(11):448. 10.3390/jcm7110448 PMC6262322 30463197

[66] Sun Y Xu H Li Z Han J Song W Wang J. Robotic Versus Laparoscopic Low Anterior Resection for Rectal Cancer: A Meta-Analysis. World J Surg Oncol (2016) 14(1):61. 10.1186/s12957-016-0816-6 26928124 PMC4772524

[67] Jones K Qassem MG Sains P Baig MK Sajid MS. Robotic Total Meso-Rectal Excision for Rectal Cancer: A Systematic Review Following the Publication of the ROLARR Trial. World J Gastrointest Oncol (2018) 10(11):449–64. 10.4251/wjgo.v10.i11.449 PMC6247103 30487956

[68] Hiranyakas A Da Silva G Wexner SD Ho Y-H Allende D Berho M. Factors Influencing Circumferential Resection Margin in Rectal Cancer. Colorectal Dis (2013) 15(3):298–303. 10.1111/j.1463-1318.2012.03179.x 22776435

[69] Warrier SK Kong JC Guerra GR Chittleborough TJ Naik A Ramsay RG. Risk Factors Associated With Circumferential Resection Margin Positivity in Rectal Cancer: A Binational Registry Study. Dis Colon Rectum (2018) 61(4):433–40. 10.1097/DCR.0000000000001026 29521824

[70] Nagtegaal ID Marijnen CAM Kranenbarg EK Van De Velde CJH Van Krieken JHJM. Circumferential Margin Involvement Is Still an Important Predictor of Local Recurrence in Rectal Carcinoma: Not One Millimeter But Two Millimeters is the Limit. Am J Surg Pathol (2002) 26(3):350–7. 10.1097/00000478-200203000-00009 11859207

[71] Helbach MV Koedam T Knol J Diederik A Spaargaren G Bonjer H. Residual Mesorectum on Postoperative Magnetic Resonance Imaging Following Transanal Total Mesorectal Excision (TaTME) and Laparoscopic Total Mesorectal Excision (LapTME) in Rectal Cancer. Surg Endoscopy (2019) 33(1):94–102. 10.1007/s00464-018-6279-9 PMC6336750 29967990

[72] Kitz J Fokas E Beissbarth T Ströbel P Wittekind C Hartmann A. Association of Plane of Total Mesorectal Excision With Prognosis of Rectal Cancer: Secondary Analysis of the CAO/ARO/AIO-04 Phase 3 Randomized Clinical Trial. JAMA Surg (2018) 153(8):e181607–e181607. 10.1001/jamasurg.2018.1607 29874375 PMC6142959

[73] Larsen SG Pfeffer F Kørner H. Norwegian Colorectal Cancer Group. Norwegian Moratorium on Transanal Total Mesorectal Excision. Br J Surg (2019) 106(9):1120–1. 10.1002/bjs.11287 31304578

[74] Bonjer HJ Deijen CL Abis GA Cuesta MA van der Pas MHGM de Lange-de Klerk ESM. A Randomized Trial of Laparoscopic Versus Open Surgery for Rectal Cancer. N Engl J Med (2015) 372(14):1324–32. 10.1056/NEJMoa1414882 25830422

[75] Wasmuth HH Faerden AE Myklebust TÅ Pfeffer F Norderval S Riis R. Transanal Total Mesorectal Excision for Rectal Cancer has Been Suspended in Norway. Br J Surg (2020) 107(1):121–30. 10.1002/bjs.11459 31802481

[76] Rullier A Gourgou-Bourgade S Jarlier M Bibeau F Chassagne-Clément C Hennequin C. Predictive Factors of Positive Circumferential Resection Margin After Radiochemotherapy for Rectal Cancer: The French Randomised Trial ACCORD12/0405 PRODIGE 2. Eur J Cancer (2013) 49(1):82–9. 10.1016/j.ejca.2012.06.028 22909998

